# Optical absorption, induced bleaching, and photoluminescence of CdSe nanoplatelets grown in cadmium octanoate matrix

**DOI:** 10.1186/1556-276X-9-88

**Published:** 2014-02-20

**Authors:** Alina Lyashchova, Andriy Dmytruk, Igor Dmitruk, Gertruda Klimusheva, Tetyana Mirnaya, Vitaliy Asaula

**Affiliations:** 1 Institute of Physics of National Academy of Sciences of Ukraine, Nauky prosp., 46, Kyiv 03028, Ukraine; 2 Faculty of Physics, Taras Shevchenko National University of Kyiv, Academician Glushkov prosp., 2, Kyiv 03022, Ukraine; 3 V. I. Vernadsky Institute of General and Inorganic Chemistry of National Academy of Sciences of Ukraine, Academician Paladin prosp., 32-34, Kyiv 03142, Ukraine

**Keywords:** Optical absorption, Photoluminescence, CdSe nanoplatelets

## Abstract

CdSe nanoparticles (NPs) are chemically synthesized in thermotropic ionic liquid crystalline (LC) phase of cadmium octanoate that was used as a nanoreactor. The nanocomposite samples are obtained by the rapid cooling of the LC phase to room temperature. Observed doublet structure in absorption spectra of the nanocomposites is characteristic for the two-dimensional CdSe nanoplatelets (NPLs). The thicknesses of the CdSe NPLs are 1.6, 1.9 and 2.3 nm as determined from the absorption spectra, and correspond to 4, 5 and 6 CdSe monolayers, respectively. Induced simultaneous bleaching of the doublet components observed under femtosecond laser excitation, as well as photoluminescence spectra and their kinetics are found compatible with the model of excitons with heavy- and light-hole valence bands confined in nanoplatelets.

## Background

Many representatives of metal alkanoate salts form the thermotropic ionic liquid crystalline phase that has the structure of the smectic A at relatively high temperatures (about +100°C and higher). The mesophase of metal alkanoates can be used as a nanoreactor for synthesis and stabilization of semiconductor and metal NPs with small dispersion of their sizes. The LC mesophase of pure metal alkanoates, as well as LC mesophase of nanocomposites with NPs, can be supercooled that leads to the subsequent formation of an anisotropic glass at the room temperature, in which the layered structure of the smectic A phase is retained [1].

Earlier, structural and optical properties of cadmium alkanoate composites with CdS quantum dots have been studied and it was shown that the template-controlled synthesis of semiconductor CdS in metal alkanoate matrix is very promising in creating nanocrystals with small dispersion of their sizes and uniformity on their shapes [2,3]. They are new perspective materials for many applications including lasers and sensors of near-ultraviolet and blue visible spectral range. It has been found that the thermo-optical nonlinearity of cadmium octanoate composites containing CdSe NPs are characterized by extremely large value of the nonlinear refractive index, n_2_, under relatively low-powered CW laser irradiation [4].

As for colloids, progress in synthesis has resulted in methods of formation of CdSe nanostructures with the atomic precision, namely, magic-sized clusters of exact number of constituting atoms [5] and CdSe nanoplatelets with two-dimensional electronic structure [6,7]. In the present paper, we discuss optical absorption and photoluminescence properties of CdSe nanocomposites prepared in cadmium octanoate matrix.

## Methods

The cadmium octanoate (Cd^+2^(C_7_H_15_COO)_2_^-^, the abbreviation CdC_8_) exists in a form of the polycrystalline powder at room temperature. The smectic A mesophase of the cadmium octanoate occurs in the temperature range 98°C to 180°C. CdSe nanoparticles (NPs) are synthesized in cadmium octanoate matrix by the following manner [4]: The polycrystalline powder of CdC_8_, impregnated with a saturated aqueous-alcoholic solution of the selenourea (starting amount of selenourea is 4 mol%), was held in a furnace (at 100°C, 180°C, or 220°C) in argon atmosphere for 30 min. The size and shape of the CdSe NPs were determined by a certain condition of the synthesis. The synthesized nanocomposites were cooled down to room temperature. As the result, the colored polycrystalline powders of CdC_8_ with CdSe NPs were obtained. As follows, from the experiments described below, CdSe NPs synthesized in CdC_8_ at various temperatures (100°C, 180°C, and 220°C) have different sizes.

The samples of glassy nanocomposites are prepared by the following method: The polycrystalline powder of the nanocomposite was placed between two flat quartz substrates. The thickness of the sample was set by a polytetrafluoroethylene stripe (10 or 30 μm). Such cell was heated up to the temperatures of the mesophase. After that, the cell was rapidly cooled down to room temperature, forming the anisotropic glassy nanocomposite [3].

### Details of TEM studies of the samples will be published elsewhere

The absorption spectrum measurements of the CdSe NPLs were carried out with the automated spectral complex KSVU-6 (LOMO). High optical quality of the samples resulted in low scattering level, and allowed us to neglect the scattering.

Measurements of photoluminescence (PL) and PL excitation (PLE) spectra of the nanocomposites were performed by spectrometer, which consisted of two monochromators (LOMO), 100-W tungsten halogen lamp, a photomultiplier tube, and necessary electronics controlled by PC. GaN laser excitation (CW, 406 nm, 75 mW) was employed also for measurements of PL spectra.

For PL kinetics, studies in nano-microsecond time interval, N_2_ pulsed laser excitation (337 nm, 6 ns, 20 Hz repetition rate, approximately 1 mJ of energy in a pulse) was used. RIGOL DS5202MA digital storage oscilloscope (200 MHz, 1GS/s) acquired signal directly from the PMT, digitized it, fitted the data by exponential decay curve, and, optionally, transferred digitized data to PC for advanced data processing.

Pump-probe measurements of transient absorption were performed at the Center for collective use ‘Laser Femtosecond Complex’ at the Institute of Physics of NASU [8]. The pump pulse parameters were the following: 400 nm, 130 fs, 1 kHz, approximately 10 μJ. The probe pulse was ‘white continuum’ generated in LiF or sapphire plate. The pump and the probe pulses overlapped on the sample. Transient spectrum of the probe was measured by Acton Research SP2500i spectrometer (Princeton Instruments, Trenton, NJ, USA) equipped with a Spec 10 CCD detector.

## Results and discussion

**Figure 1:**
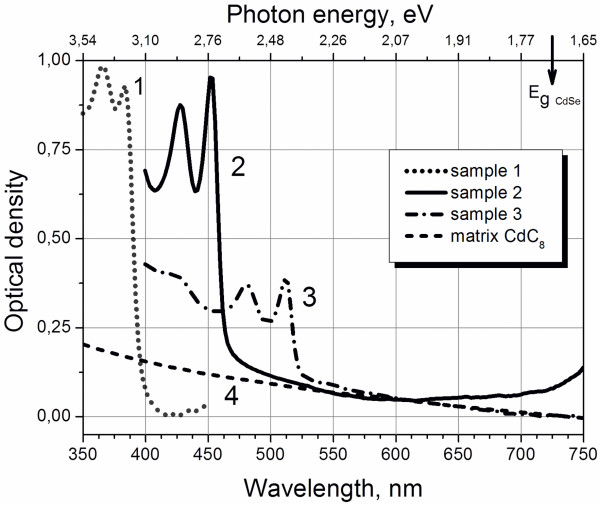
**Absorption spectra.** Synthesized CdSe NPs in cadmium octanoate matrix (curves 1, 2, 3). The CdC_8_ matrix does not absorb light in visible spectral region (curve 4).

The absorption spectra of the CdSe NPs synthesized at different temperatures (100°C, 180°C, and 220°C, thereafter called ‘sample 1’, ‘sample 2’, and ‘sample 3’) in cadmium octanoate matrix are shown in Figure 1.

The doublets in the absorption spectra prompt to suppose the nanoplatelet shape of the formed CdSe nanoparticles, as it was proposed in the paper [6]. The absorption bands at 366 nm (3.390 eV) and 384 nm (3.221 eV) of sample 1, 430 nm (2.883 eV) and 454 nm (2.731 eV) of sample 2, as well as the bands at 483 nm (2.567 eV) and 514 nm (2.412 eV) of sample 3 can be associated with electron transitions from light-hole (LH) and heavy-hole (HH) energy levels of valence band into the lowest energy level of conduction band, respectively [6,7]. Corresponding excitons in bulk crystals are known also as B- and A-excitons, respectively.

**Figure 2:**
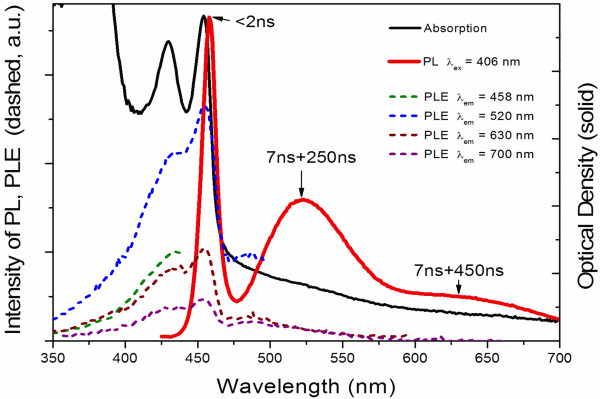
Optical absorption, photoluminescence, and photoluminescence excitation spectra of sample 2.

In the effective mass approximation the Schrödinger equation was solved for a rectangular symmetrical potential well, which has a finite depth *U*_0_[9]. The expression for the energy as a function of the size of the well was obtained for electrons and holes separately: *E*_e_(*a*), *E*_LH_(*a*), *E*_HH_(*a*). The difference between the energy of the absorption edge of the matrix *E*_*matrix*_ and the band gap energy of CdSe bulk crystal *E*_*g*_ determines the depth of the quantum well. The energy of the exciton absorption is defined as *E*_1_ = *E*_e_ + *E*_HH_ + *E*_g_ (*E*_1_ corresponds to the lower energy peak in the absorption doublet in Figure 1) and *E*_2_ = *E*_e_ + *E*_LH_ + *E*_g_ (*E*_2_ corresponds to the higher energy peak in the doublet). For the calculations, we used the following values: *E*_*matrix*_ = 5.5 eV (determined from absorption spectra), *E*_g_ = 1.7 eV, effective mass of electron *m*_e_ = 0.11*m*_o_ (where *m*_o_ is the free-electron mass) [10]. Ithurria et al. used the following set of effective masses for quasi-two-dimentional CdSe NPLs: *m*_LH_ = 0.19*m*_o_ (for light hole) and *m*_HH_ = 0.89*m*_o_ (for heavy hole). These parameters were adapted to the experimental results on CdSe NPLs with a cubic crystal structure. Our adapted parameters to experimental values are the following: *m*_LH_ = 0.41*m*_o_, *m*_HH_ = 0.92*m*_o_. Considering the NPL as a quantum well, its thickness was estimated from the position of the excitonic peak in the absorption spectrum. The calculated thicknesses are listed in Table 1. These values are slightly larger than the thicknesses of CdSe NPLs with cubic structure obtained previously [6,7]. This fact may indicate other crystal structure of our NPLs synthesized in cadmium octanoate matrix. The PL and PLE spectra of sample 2 are presented in Figure 2. PL spectrum, measured by 406-nm laser excitation, consists of a sharp peak at 458 nm (2.707 eV), a broad band centered at 520 nm (2.38 eV) and long-wavelength shoulder at about 630 nm (1.97 eV). The sharp peak almost overlaps with the absorption band 454 nm (2.731 eV). It corresponds to free eHH-exciton (electron-HH) recombination in the volume of CdSe NPLs. The band at 520 nm and the long-wavelength shoulder can be connected with recombination of localized excitons at the surface of the NPLs. The different wavelengths of 520 and 630 nm bands, that accompany the recombination of localized excitons, indicate their localization at different sites of the NPL surface, which may be associated with the flat surfaces and the end surface of the NPLs.PL decay times shown in Figure 2 are pointed at the wavelengths, where they have been measured. The mono-exponential fast decay of the short-wavelength PL (<2 ns at 458 nm) supports its assignment to the free eHH-exciton recombination. The slow and bi-exponential character of the long-wavelength PL decay (7 and 250 ns at 520 nm, and 7 and 450 ns at 630 nm) definitely supports the suggestion of corresponding exciton localization. The bi-exponential decay kinetics also indicates the existence of different sites for such localization at NPL surface.PLE spectra measured for different PL wavelengths have similar structure but different intensity (Figure 2, dashed curves), indicating that all the PL bands should be associated with radiative transitions in the same objects (NPLs), and excluding a possibility of NPL dispersion of shape or structure. A PLE spectrum consists of a sharp peak at 454 nm and much weaker peak (or even shoulder) at about 434 nm. The two short-wavelength peaks obviously correspond to the two peaks of optical absorption spectrum, which we assign to electron-HH and electron-LH transitions. Despite the similarity of PLE and absorption spectra, there is an obvious disparity between them; intensities of the peaks of electron-HH and electron-LH transitions in these spectra are significantly different: electron-LH transition is much less pronounced in PLE spectrum than in the absorption spectrum. This disparity suggests an existence of a nonradiative relaxation channel of eLH-excitons in the NPLs beside the main their relaxation via eHH-exciton radiative recombination.

**Table 1:** Calculated thicknesses of CdSe NPLs

**Sample**	**Thickness (nm)**		**Supposed number of CdSe monolayers**
	**From eHH-exciton band position**	**From eLH-exciton band position**	
**1**	1.34	1.34	4
**2**	1.70	1.70	5
**3**	2.04	2.03	6

**Figure 3:**
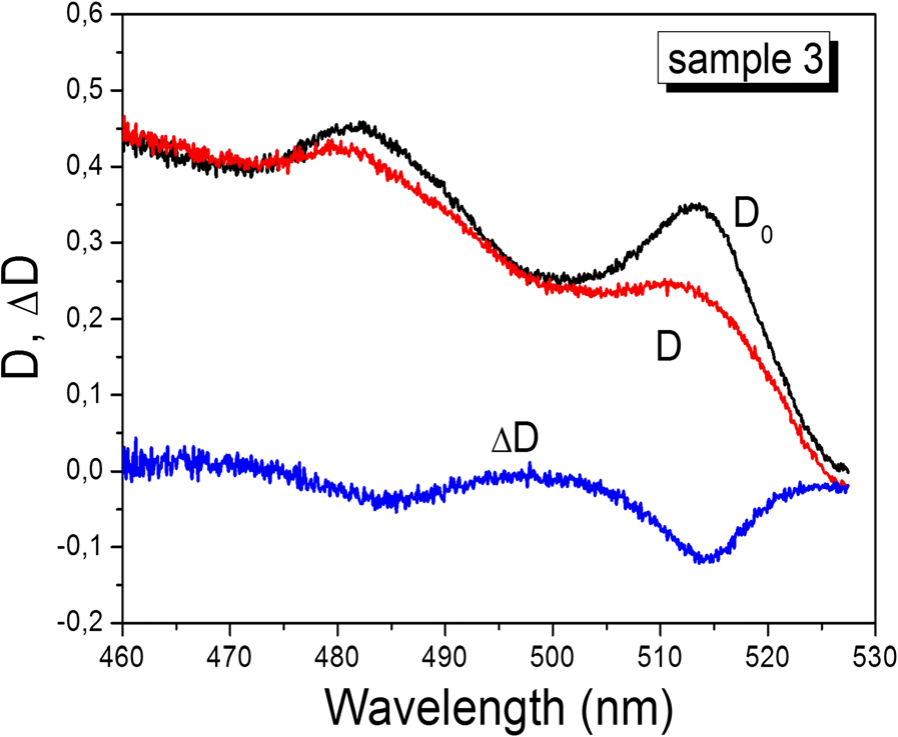
**Optical density of CdSe NPLs in cadmium octanoate matrix.** Before (curve D_0_) and under (curve D) the irradiation with pump pulse, and their difference (curve **Δ**D).

PL spectra of samples 1 and 3 demonstrate in general a similar structure to those described above with the main transitions corresponding to recombination of eHH-excitons.The change of optical density of the eHH- and eLH-exciton bands of sample 3 has been investigated by pump-probe technique in the femto-picosecond time interval (Figure 3). Usually for semiconductor nanoparticles, optical density decreases under the action of high-intensity pump pulse, i.e., transient induced bleaching is observed. It is found that both components of the absorption doublet, associated with electron transitions from LH and HH energy levels, demonstrate the induced bleaching simultaneously. That again supports their assignment to the same object.

## Conclusions

Formation of the nanoplatelet shape CdSe nanoparticles in the thermotropic ionic liquid crystalline phase of cadmium octanoate is confirmed by the doublet in the absorption spectrum, simultaneous to the induced bleaching of its components, as well as by photoluminescence properties. The two sharp peaks of optical absorption can be associated with electron transitions from light-hole and heavy-hole energy levels of valence band into the lowest energy level of conduction band. Thanks to the large oscillator strength of optical transitions and huge nonlinearity, these CdSe NPL nanocomposites are new perspective materials for many applications.

## Competing interests

The authors declare that they have no competing interests.

## Authors’ contributions

TM and VA synthesized the CdSe nanoparticles in cadmium octanoate matrix. GVK carried out the preparation of the samples. IMD and AMD carried out the design of the luminescence study and properties of optical absorption. AL made calculations. GVK, AMD, IMD, and AL did the in-depth analysis and drafted this manuscript. All authors read and approved the final manuscript.
